# Association of Mitral Valve Geometry at CT with Secondary Mitral Regurgitation after Transcatheter Aortic Valve Replacement in Patients with Aortic Regurgitation

**DOI:** 10.31083/j.rcm2507241

**Published:** 2024-07-02

**Authors:** Minyan Yin, Yuntao Lu, Xue Yang, Lili Dong, Xiaolin Wang, Lai Wei

**Affiliations:** ^1^Shanghai Institute of Medical Imaging, 200032 Shanghai, China; ^2^Department of Radiology, Zhongshan Hospital, Fudan University, 200032 Shanghai, China; ^3^Department of Cardiovascular Surgery, Zhongshan Hospital, Fudan University, 200032 Shanghai, China; ^4^Shanghai Engineering Research Center of Heart Valve, 200032 Shanghai, China; ^5^Department of Echocardiography, Zhongshan Hospital, Fudan University, 200032 Shanghai, China; ^6^Department of Interventional Radiology, Zhongshan Hospital, Fudan University, 200032 Shanghai, China

**Keywords:** mitral valve, computed tomography, secondary mitral regurgitation, aortic regurgitation, transcatheter aortic valve replacement

## Abstract

**Background:**

The improvement rate and predictors of secondary mitral regurgitation in patients with aortic regurgitation undergoing transcatheter aortic valve replacement (TAVR) remain unclear. This study aimed to identify predictors of persistent moderate to severe secondary mitral regurgitation after TAVR in patients with aortic regurgitation by assessing mitral valve geometry with computed tomography (CT).

**Methods:**

This retrospective cohort study reviewed 242 consecutive patients with aortic regurgitation who underwent TAVR between May 2014 and December 2022. Patients with primary or less than moderate mitral regurgitation were excluded. Mitral annular dimensions (area, perimeter, anteroposterior, intercommissural, and trigone-to-trigone diameter), mitral valve tenting geometry (mitral valve tenting area [MVTA] and mitral valve tenting height [MVTH]), and papillary muscle displacement were systematically measured at CT. Mitral regurgitation improvement was assessed at 3 months after TAVR by echocardiography. Logistic regression was performed to explore the association of mitral valve geometry with mitral regurgitation improvement after TAVR.

**Results:**

A total of 75 patients (mean age, 74 ± 7 years; 32.0% female) with moderate to severe secondary mitral regurgitation were included in the final analysis. Mitral regurgitation improved in 49 patients and remained unchanged in 26 patients. Mitral annular dimensions, including area, perimeter, anteroposterior, and intercommissural diameter, were associated with mitral regurgitation improvement. MVTA and MVTH were risk factors for sustained mitral regurgitation. In addition, QRS duration >120 ms and atrial fibrillation had an impact on the mitral regurgitation improvement. Mitral annular area (odds ratio [OR], 1.41; 95% confidence interval [CI]: 1.05, 1.90; *p* = 0.02) and MVTA (OR, 7.24; 95% CI: 1.72, 30.44; *p* = 0.007) were independent predictors of persistent secondary mitral regurgitation after TAVR.

**Conclusions:**

Mitral annular area and MVTA were independent predictors of persistent secondary mitral regurgitation after TAVR.

## 1. Introduction

Transcatheter aortic valve replacement (TAVR) has matured into a well-established therapeutic option for symptomatic aortic stenosis [1, 2]. Moreover, the advent of novel transcatheter heart valves designed for pure aortic regurgitation has led to an increasing trend of off-label use of TAVR in patients suffering from severe aortic regurgitation and prohibitive surgical risk [3, 4]. Notably, coexistent moderate to severe mitral regurgitation is common in patients with hemodynamically-significant aortic regurgitation, with prevalence ranging from 14% to 25% [5, 6]. Secondary mitral regurgitation is more frequently observed than primary mitral regurgitation among these patients, indicating an advanced stage in clinical spectrum of aortic regurgitation and predicting a worse clinical outcome [6].

Previous studies have shown significant improvement in secondary mitral regurgitation following TAVR in patients with aortic stenosis, with improvement rates even as high as 80% [7, 8]. Additionally, the size of mitral annular is related to mitral regurgitation improvement [7]. However, little is known about improvement rates of secondary mitral regurgitation after TAVR in patients with aortic regurgitation, as well as predictors of mitral regurgitation improvement. Transcatheter mitral valve repair has emerged as the standard invasive treatment for patients with severe symptomatic secondary mitral regurgitation, with a growing number of research showing improved prognosis and cardiovascular events [9, 10]. Hence, transcatheter mitral valve repair could be performed along with TAVR in selected patients with moderate to severe secondary mitral regurgitation that is expected to persist after TAVR. Therefore, it is necessary to determine whether there are preoperative indicators of persistent secondary mitral regurgitation after TAVR.

Secondary mitral regurgitation is characterized by the preservation of normal leaflet and tendinous cords, but deformation and remodeling of the valve and subvalvular apparatus [11]. Cardiac computed tomography (CT) is of great value for the evaluation of valve anatomy and has become essential to TAVR preoperative planning [12]. Nevertheless, no research has yet examined discrepancies in mitral valve complex at CT between improved and persistent secondary mitral regurgitation after TAVR. We therefore sought to seek predictors of sustained moderate to severe secondary mitral regurgitation after TAVR in patients with aortic regurgitation through granular assessment of the mitral valve apparatus by cardiac CT, combined with clinical indicators.

## 2. Materials and Methods 

### 2.1 Study Population 

**Fig. 1.**
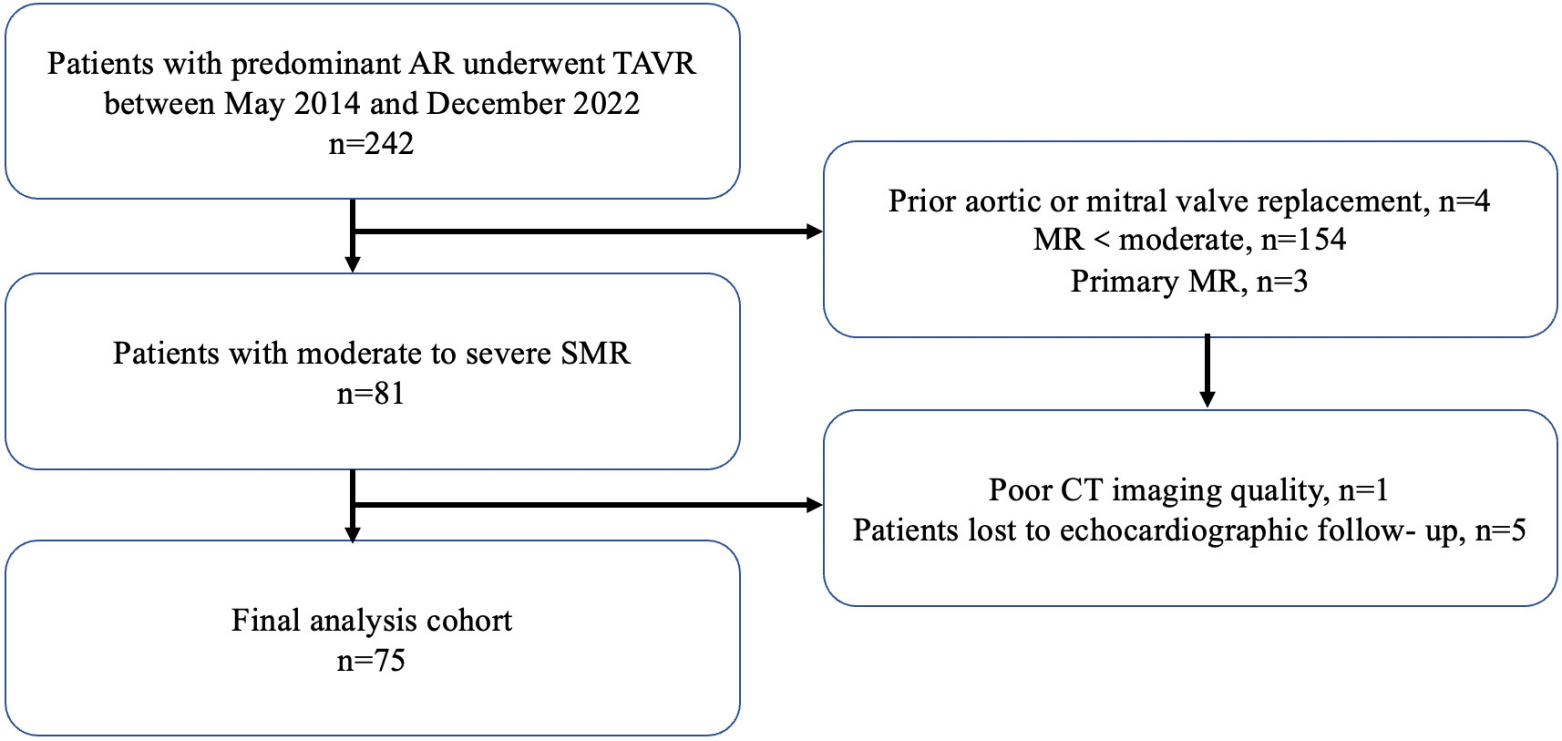
**Flowchart of the study.** AR, aortic regurgitation; TAVR, transcatheter aortic valve replacement; MR, mitral regurgitation; SMR, secondary mitral regurgitation; CT, computed tomography.

This retrospective study included 242 consecutive patients who underwent TAVR for severe symptomatic aortic regurgitation at one center from May 2014 to December 2022. The Ethics Committee of the Zhongshan Hospital, Fudan University approved this study (KY2023239, March 31, 2023) with waiver of informed consent. All cases were manually reviewed to determine eligibility. Exclusion criteria included: (1) patients with prior aortic or mitral valve replacement; (2) patients diagnosed with primary mitral regurgitation and those with less than moderate secondary mitral regurgitation; (3) patients with poor CT image quality or lost to echocardiographic follow-up. A total of 75 qualified patients were included in the final analysis (Fig. 1). The study population was divided into 2 groups according to their mitral regurgitation improvement assessed by echocardiography at 3 months after TAVR: improved mitral regurgitation (IMR, the severity of mitral regurgitation was reduced by at least one grade) and nonimproved mitral regurgitation (NMR, less or no improvement in mitral regurgitation).

### 2.2 Echocardiography 

All patients underwent complete 2-dimensional and color Doppler echocardiography at baseline and at 3 months after TAVR. Analysis of the degree and etiology of mitral regurgitation was performed by a sole echocardiographer with more than 10 years of experience, blinded to further data on the postoperative outcomes. Mitral regurgitation was classified into primary (intrinsic mitral valve lesions resulting in mitral regurgitation) and secondary (mitral regurgitation caused by mitral valve deformation or remodeling). The severity of mitral regurgitation was graded as none, trivial, mild, moderate, and severe by using a multiparametric approach that included the assessment of regurgitant jet width and area, together with left ventricular (LV) and left atrial (LA) dimensions. LV ejection fraction was measured using the biplane disks method.

### 2.3 CT Data Acquisition and Analysis 

**Fig. 2.**
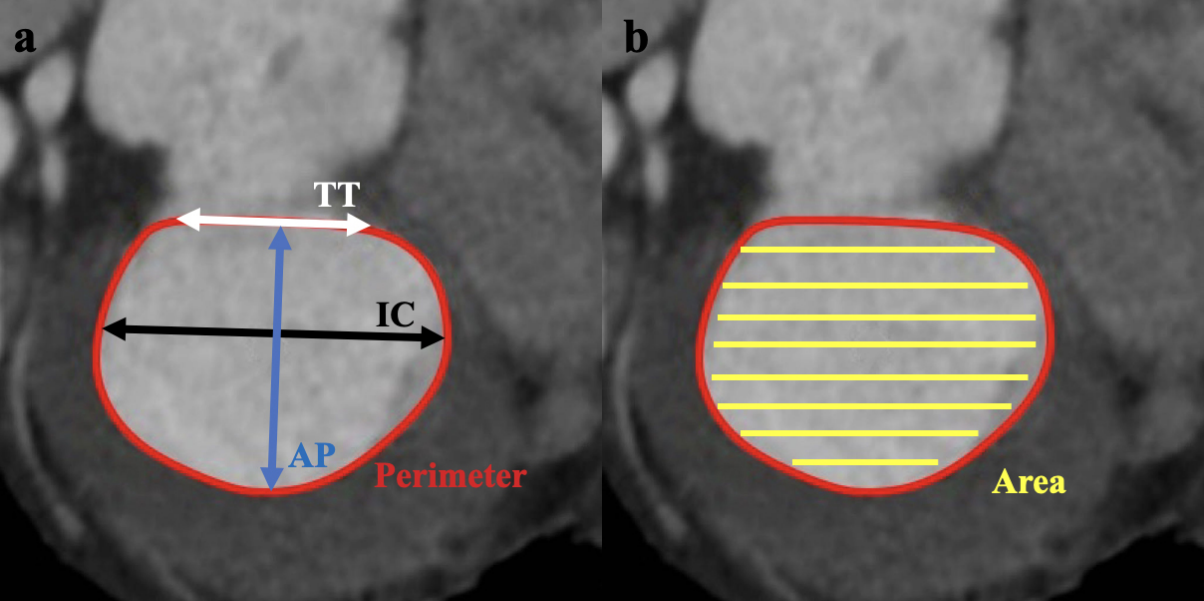
**D-shaped mitral annular analysis.** (a) Measurement of the annular perimeter (red outline), intercommissural (IC, black arrow), anteroposterior (AP, blue arrow), and trigone-trigone (TT, white arrow) distances. (b) Measurement of the annular area (yellow shadow). IC, intercommissural; AP, anteroposterior; TT, trigone-trigone.

**Fig. 3.**
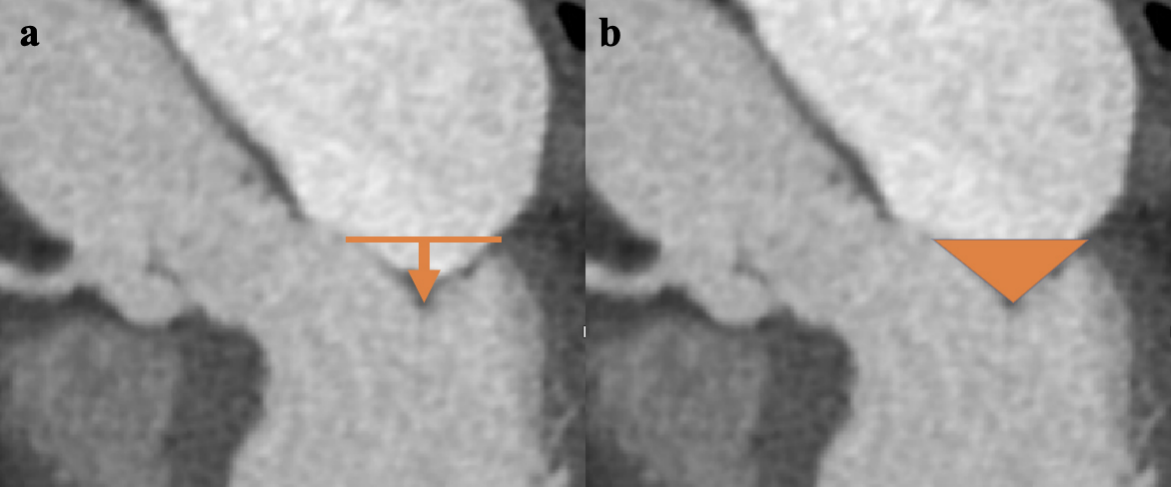
**Mitral valve tenting analysis.** (a) Measurement of MVTH (orange arrow). (b) Measurement of MVTA (orange triangle). MVTH, mitral valve tenting height; MVTA, mitral valve tenting aera.

**Fig. 4.**
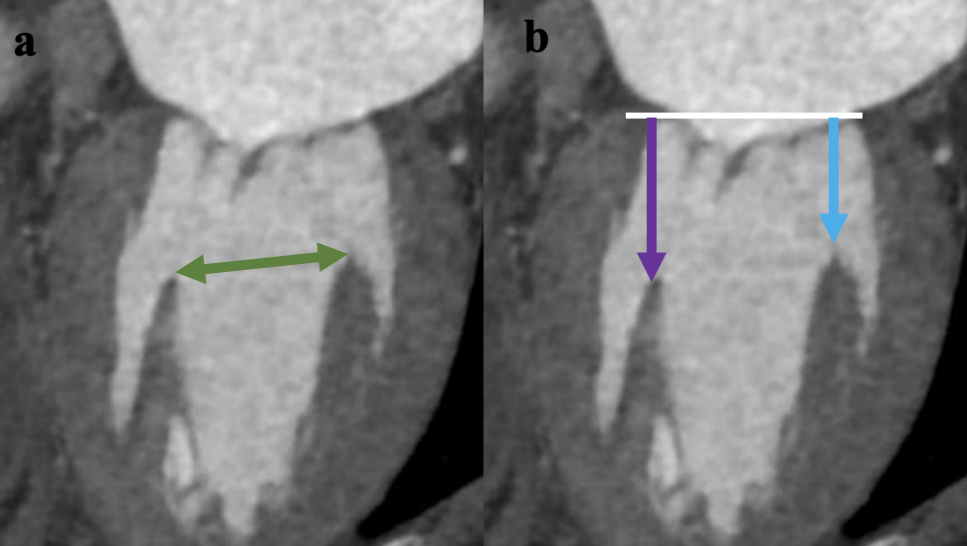
**Papillary muscle displacement analysis.** (a) Measurement of the distance between the heads of papillary muscles (green arrow). (b) Measurement of the distance from the annulus to posteromedial papillary muscle (purple arrow) and anterolateral papillary muscle (blue arrow).

Cardiac CT was obtained using either a first- or second-generation dual-source CT scanner (Definition, Siemens Healthineers, Erlangen, Germany). Tube voltage and current were adjusted to body habitus using retrospective electrocardiogram (ECG)-gated data acquisition. For contrast-enhanced data acquisition, approximately 80 mL of contrast agent (370 mg iodine/mL, Ultravist, Bayer, Leverkusen, Germany) was injected into antecubital vein using a biphasic injection method (contrast agent and saline). The scan range extended from the neck to the diaphragm. Axial images were reconstructed at 10% intervals of the cardiac cycle. The evaluation of mitral valve geometry was performed at end-systolic and offline on a dedicated valve analysis workstation (3mensio Structural Heart V7.0, Pie Medical Imaging, Maastricht, The Netherlands). The simplified Dshaped mitral annulus was used to evaluate the annular dimensions in this study. The segmentation of the D-shaped mitral annular was performed as previously described [13]. The annular area, perimeter, trigone-to-trigone, anteroposterior and intercommissural diameter were measured (Fig. 2). Mitral valve tenting height (MVTH) and mitral valve tenting area (MVTA) were measured on the 3-chamber view (Fig. 3). MVTH was indicated as the vertical distance between the mitral annular plane and the coaptation of mitral leaflets. MVTA was defined as the area enclosed by the anterior and posterior mitral valve leaflets and annulus. Papillary muscle displacement was analysed as shown in Fig. 4. The distance from the annulus to the posteriormedial papillary muscle and the anterolateral papillary muscle, and the distance between the heads of the papillary muscles, were measured separately. All CT measurements were initially performed by an observer with 2 years of experience in cardiac CT imaging, blinded to patient identifying information. In order to analyze the reproducibility of the measurements, 20 patients were randomly selected from the cohort. All parameters were measured by the same observer and a second observer with 9 years of experience in cardiac CT imaging a week apart.

### 2.4 Statistical Analysis 

Normally distributed continuous variables were expressed as mean ± standard deviation and compared with Student *t* test. Non-normally distributed continuous variables were presented as medians [25th to 75th interquartile range] and compared with Mann-Whitney U test. Categorical variables were expressed in numbers with percentages and compared with the chi-square test or continuity-corrected chi-square test. Logistic regression was performed to explore the association between mitral valve geometry at CT and less mitral regurgitation improvement at 3 month after TAVR. Variables with statistically significant in the univariable analysis and known risk factors for less mitral regurgitation improvement from literature were included in the multivariable logistic regression model to identify independent predictors of less mitral regurgitation improvement. A two-tailed *p*-value < 0.05 was considered as statistically significant. The results were presented as odds ratios (ORs) with corresponding 95% confidence intervals (CIs). Receiver operator characteristic curve analysis was performed to determine the predictive value. Intraobserver and interobserver agreement were assessed using intraclass correlation coefficients. All statistical analyses were performed using SPSS software (version 25.0; IBM Corp, Armonk, NY, USA).

## 3. Results 

### 3.1 Baseline Characteristics 

The final cohort study included 75 patients with moderate to severe secondary mitral regurgitation who underwent TAVR for aortic regurgitation (mean age, 74 ± 7 years; 32.0% female). Among them, 49 patients (65.3%) with at least one grade reduction in mitral regurgitation severity at 3 months after TAVR were enrolled in IMR and 26 patients (34.7%) with less or no improvement in mitral regurgitation were admitted to NMR. Baseline clinical characteristics are summarized in Table 1. Compared with the IMR group, the QRS duration >120 ms was observed more frequently in the NMR group (8 of 49 [16.3%] *vs.* 10 of 26 [38.5%]; *p* = 0.033). In addition, the proportion of atrial fibrillation was higher in the NMR group than IMR group (9 of 26 [34.6%] *vs.* 7 of 49 [14.3%]; *p* = 0.041). No significant differences were found between the two groups in terms of LA and LV sizes, as well as LV ejection fraction. However, patients in the NMR group had relatively larger LA and LV dimensions and a lower LV ejection fraction compared to those in the IMR group. Similarly, other characteristics, including age, gender, New York Heart Association functional class, Society of Thoracic Surgeons score, coronary artery disease and other comorbidities showed no differences between NMR and IMR.

**Table 1.** **Baseline characteristics**.

			Total (n = 75)	IMR (n = 49)	NMR (n = 26)	*p* value
Clinical variables						
	Age (years)		74 ± 7	74 ± 6	74 ± 8	0.761
	Female		24 (32.0)	15 (30.6)	9 (34.6)	0.724
	BMI (${\mathrm{kg/m}}^{2}$)		22.5 ± 5.2	22.7 ± 6.1	22.2 ± 2.9	0.726
	NYHA functional class					0.657
		Class II	15 (20.0)	10 (20.4)	5 (19.2)	
		Class III	51 (68.0)	34 (69.4)	17 (65.4)	
		Class IV	9 (12.0)	5 (10.2)	4 (15.4)	
	STS (%)		4.2 (2.4–6.7)	4.0 (2.5–6.8)	4.4 (2.4–6.7)	0.947
	Hypertension		53 (70.7)	32 (65.3)	21 (80.8)	0.162
	Diabetes mellitus		12 (16.0)	8 (16.3)	4 (15.4)	1.000
	COPD		17 (22.7)	14 (28.6)	3 (11.5)	0.094
	Coronary artery disease		13 (17.3)	8 (16.3)	5 (19.2)	1.000
	Peripheral vascular disease		16 (21.3)	11 (22.4)	5 (19.2)	0.746
	Previous cardiac surgery		3 (4.0)	2 (4.1)	1 (3.8)	1.000
	Creatinine (mg/dL)		1.4 ± 1.0	1.2 ± 0.5	1.6 ± 1.5	0.246
Electrocardiogram characteristics						
	Atrial fibrillation		16 (21.3)	7 (14.3)	9 (34.6)	0.041
	QRS duration >120 ms		18 (24.0)	8 (16.3)	10 (38.5)	0.033
Echocardiographic characteristics						
	LA (mm)		47 ± 7	46 ± 6	49 ± 8	0.053
	LVESD (mm)		49 ± 10	48 ± 9	52 ± 10	0.050
	LVEF (%)		46 ± 12	47 ± 13	42 ± 9	0.052
	PASP (mmHg)		44 ± 12	44 ± 13	42 ± 9	0.488
	Moderate-severe TR		22 (29.3)	14 (28.6)	8 (30.8)	0.842

Values are mean ± standard deviation, median [25th to 75th interquartile range], or frequency (percentage). IMR, improved mitral regurgitation; NMR, nonimproved mitral regurgitation; BMI, body mass index; NYHA, New York Heart Association; STS, Society of Thoracic Surgeons Predicted Risk of Mortality; COPD, chronic obstructive pulmonary disease; LA, left atrial; LVESD, left ventricular end-systolic dimension; LVEF, left ventricular ejection fraction; PASP, pulmonary artery systolic pressure; TR, tricuspid regurgitation.

### 3.2 Mitral Valve Geometry 

The geometric features of the mitral valve evaluated by CT are shown in Table 2. The area and perimeter of the D-shaped mitral annulus were significantly larger in the NMR group compared with the IMR group (11.6 ± 2.4 ${\mathrm{cm}}^{2}$ *vs.* 9.9 ± 1.9 ${\mathrm{cm}}^{2}$, *p* = 0.002; 127 ± 13 mm *vs.* 118 ± 11 mm, *p* = 0.003). In addition, both the anteroposterior and intercommissural diameter of the mitral annulus were observed longer in the NMR group (32 ± 4 mm *vs.* 29 ± 4 mm, *p* = 0.002; 43 ± 4 mm *vs.* 40 ± 4 mm, *p* = 0.014). However, the trigone-to-trigone distance was comparable in both groups. Moreover, patients in the NMR group had significantly greater MVTH and MVTA than those in the IMR group (11 ± 2 mm *vs.* 9 ± 2 mm, *p* < 0.001; 1.5 ± 0.4 ${\mathrm{cm}}^{2}$ *vs.* 1.2 ± 0.4 ${\mathrm{cm}}^{2}$, *p* = 0.001). Finally, there was no statistically significant difference in the distance between the heads of the papillary muscles or the distance from the mitral annulus to the head of the posteriormedial papillary muscle and the anterolateral papillary muscle. Reproducibility data were analyzed in 20 randomly selected patients. The intraobserver and interobserver agreement for mitral valve assessment by CT was excellent, with intraclass correlation coefficients ranging from 0.86 to 0.99 (**Supplementary Table 1**).

**Table 2.** **Mitral valve geometry**.

Variables	Total (n = 75)	IMR (n = 49)	NMR (n = 26)	*p* value
Trigone-to-trigon distance (mm)	26 ± 3	25 ± 3	26 ± 3	0.613
Anteroposterior distance (mm)	30 ± 4	29 ± 4	32 ± 4	0.002
Intercommisural distance (mm)	41 ± 4	40 ± 4	43 ± 4	0.014
Annulus area (${\mathrm{cm}}^{2}$)	10.5 ± 2.2	9.9 ± 1.9	11.6 ± 2.4	0.002
Annulus perimeter (mm)	121 ± 12	118 ± 11	127 ± 13	0.003
MVTH (mm)	10 ± 2	9 ± 2	11 ± 2	<0.001
MVTA (${\mathrm{cm}}^{2}$)	1.3 ± 0.4	1.2 ± 0.4	1.5 ± 0.4	0.001
ALPM-MA distance (mm)	22 ± 4	22 ± 5	23 ± 4	0.661
PMPM-MA distance (mm)	26 ± 5	25 ± 5	27 ± 5	0.190
PM-PM distance (mm)	23 ± 5	22 ± 5	25 ± 5	0.056

Values are mean ± standard deviation. IMR, improved mitral regurgitation; NMR, nonimproved mitral regurgitation; MVTH, mitral valve tenting height; MVTA, mitral valve tenting aera; ALPM-MA, anterolateral papillary muscle to mitral annulus; PMPM-MA, posteromedial papillary muscle to mitral annulus; PM-PM, papillary muscle head to papillary muscle head.

### 3.3 Risk Analysis for Less Improvement Mitral Regurgitation 

**Fig. 5.**
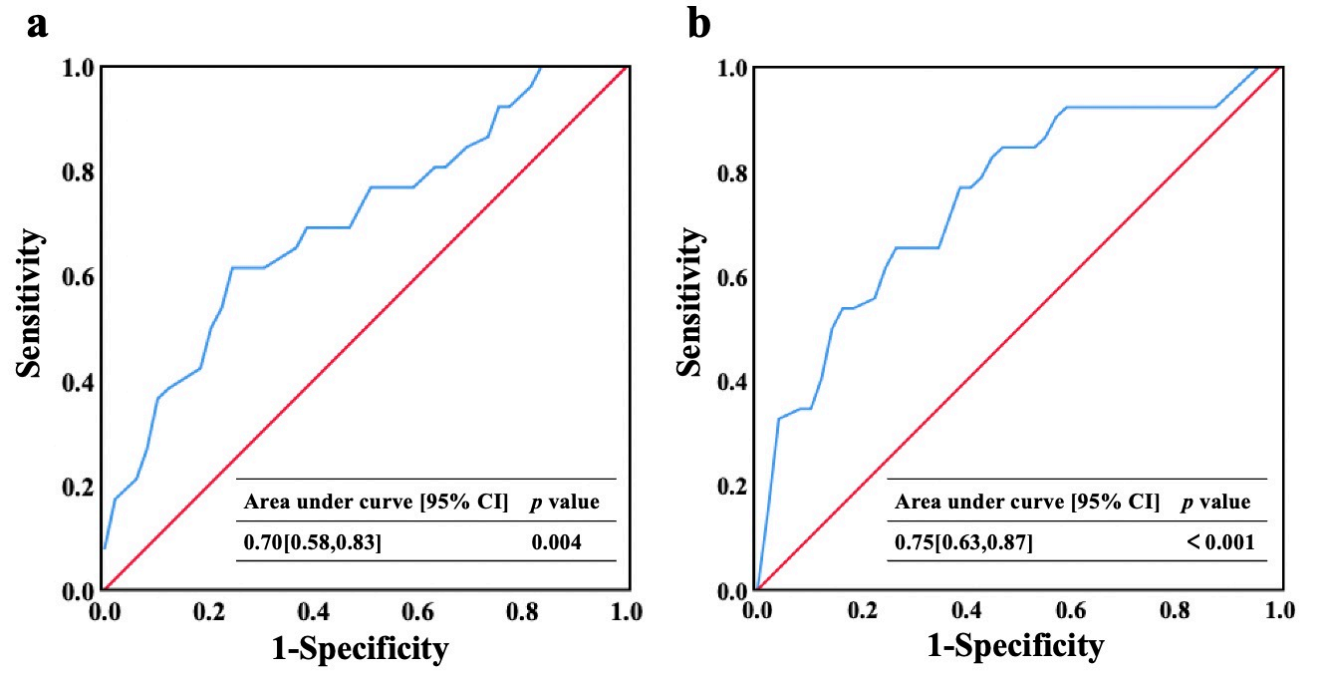
**Mitral annular area (a) and MVTA (b) predicting mitral regurgitation improvement.** MVTA, mitral valve tenting area.

In univariable analysis (**Supplementary Table 2**), larger D-shaped mitral annular dimensions, including area, perimeter, anteroposterior, and intercommissural diameter, were related to less mitral regurgitation improvement. Similarly, the greater the MVTH and MVTA, the less likely mitral regurgitation will improve. Furthermore, QRS duration >120 ms and atrial fibrillation were associated with less mitral regurgitation improvement. In multivariable analysis (Table 3), mitral annulus area (OR, 1.41; 95% CI: 1.05, 1.90; *p* = 0.022) and MVTA (OR, 7.24; 95% CI: 1.72, 30.44; *p* = 0.007) were validated as independent predictors of less mitral regurgitation improvement after TAVR, after adjustment for coronary artery disease, atrial fibrillation, QRS duration >120 ms, and LV ejection fraction. Receiver operator characteristic curves of mitral annulus area and MVTA predicting mitral regurgitation improvement were shown in Fig. 5.

**Table 3.** **Multivariable logistic regression to predict persistent mitral regurgitation after transcatheter aortic valve replacement**.

Variables		Odds ratio (95% confidence interval)	*p* value
Model 1			
	Coronary artery disease	1.14 (0.28, 4.57)	0.859
	Atrial fibrillation	1.06 (0.26, 4.28)	0.937
	QRS duration >120 ms	2.97 (0.88, 10.05)	0.080
	LVEF (%)	0.97 (0.93, 1.02)	0.267
	Mitral annular area (${\mathrm{cm}}^{2}$)	1.41 (1.05, 1.90)	0.022
Model 2			
	Coronary artery disease	0.79 (0.18, 3.37)	0.745
	Atrial fibrillation	1.67 (0.45, 6.18)	0.442
	QRS duration >120 ms	2.46 (0.71, 8.55)	0.156
	LVEF (%)	0.97 (0.93, 1.02)	0.270
	MVTA (${\mathrm{cm}}^{2}$)	7.24 (1.72, 30.44)	0.007

Values are ORs (95% CIs). LVEF, left ventricular ejection fraction; MVTA, mitral valve tenting aera; OR, odds ratio; CI, confidence interval.

## 4. Discussion 

To our knowledge, the present study is the first to use ECG-gated cardiac CT to assess the geometric differences within the mitral valve complex between improved and persistent secondary mitral regurgitation after TAVR in patients with aortic regurgitation, and to identify risk factors for persistent mitral regurgitation. In this single-center retrospective study, the proportion of improved secondary mitral regurgitation occurring at 3 months after TAVR was 65.3%, with 34.7% of patients remaining unchanged. Mitral annular area (OR, 1.41; 95% CI: 1.05, 1.90; *p* = 0.022) and MVTA (OR, 7.24; 95% CI: 1.72, 30.44; *p* = 0.007) were independent predictors of less mitral regurgitation improvement after TAVR.

The correct functioning of the mitral valve depends on the coordinated and synchronized work of its anatomic components, which in turn are tightly reliant on the function of the LV and LA [11]. Progressive, uncorrected aortic regurgitation leads to left ventricular pressure and volume overload, causing malignant left ventricular remodeling, subsequent papillary muscle displacement, mitral leaflet tethering, and mitral annular dilatation, ultimately leading to mitral regurgitation [14, 15]. In addition, an enlarged LA, especially in patients with atrial fibrillation, can cause further dilation of the mitral annular, thereby worsening mitral regurgitation [16, 17]. CT allows a comprehensive assessment of the mitral valve through precise measurement of annulus size and detailed evaluation of the subvalvular apparatus geometry [18, 19]. In addition to trigone-to-trigone distance, larger D-shaped mitral annulus measurements, including area, perimeter, anteroposterior and intercommissural distance, were associated with less mitral regurgitation improvement after TAVR in the present study. In multivariable analysis, the mitral annular area was confirmed as an independent predictor of less mitral regurgitation improvement after TAVR. Thus, a larger annulus is susceptible to sustained mitral regurgitation after TAVR. This finding is consistent with previous studies in patients with aortic stenosis [20]. The triangle-to-triangle distance denotes the distance between two triangular fibrous structures located at both ends of the base of the anterior leaflet and is relatively resistant to deformation and change [21]. Therefore, the triangle-to-triangle distance may remain unchanged or increase slightly in patients with a significantly enlarged mitral annulus, making it a poor indicator of mitral regurgitation evolution after TAVR.

In the current study, MVTA was identified as an independent risk factor for less mitral regurgitation improvement after TAVR. The extent of mitral valve tenting is indicative of the severity of secondary mitral regurgitation and has a strong correlation with the prognosis [22]. Systolic tenting was initially thought to be the consequence of LV remodeling, by displacing the papillary muscle away from the mitral annular plane, thus causing leaflet tension and tethering [23]. Advances in three-dimensional echocardiography have led to a better understanding of the complex mechanisms of secondary mitral regurgitation and introduced the concept of ‘atriogenic’ leaflet tethering caused by LA enlargement [24, 25]. Although both papillary muscle displacement and mitral valve tenting are primary markers of subvalvular remodeling in mitral regurgitation, the former is solely influenced by LV remodeling, whereas the latter is influenced by both LV and LA remodeling. This may partially explain why the degree of mitral valve tenting was associated with mitral regurgitation improvement after TAVR in this study, while the relationship of papillary muscle displacement to mitral regurgitation was not identified.

## 5. Limitations 

Limitations exist in the present study. The retrospective nature and the small sample size are major limitations. Therefore, the conclusions drawn are not sufficiently robust and need to be further verified by future prospective large sample size studies. Owing to the restricted sample size, a separate analysis of the distinct subtypes of secondary mitral regurgitation (atrial and ventricular mitral regurgitation) was unattainable. Additionally, the follow-up duration was insufficient. It is imperative to prolong the follow-up period to 6 months, 1 year, and 2 years post-TAVR in future studies to monitor the temporal evolution of mitral regurgitation. Furthermore, it is possible that the mitral valve parameters derived from CT at end-systole are over- or underestimated. As the images are reconstructed within 10% intervals of the cardiac cycle, end-systole may not be captured optimally. Finally, conventional 2-dimensional echocardiography to assess the severity of secondary mitral regurgitation is challenging, particularly in the presence of eccentric regurgitant jets or multiple regurgitant jets, resulting in an underestimation of the degree of regurgitation.

## 6. Conclusions 

In conclusion, concomitant moderate to severe secondary mitral regurgitation is common in patients with aortic regurgitation undergoing TAVR, with approximately two-thirds showing improvement post-TAVR. CT-derived mitral annular area and MVTA were independent predictors of less mitral regurgitation improvement after TAVR. Considering the unfavorable prognosis of residual secondary mitral regurgitation, these two parameters can be used as valuable references to assist decision-making regarding simultaneous performance of TAVR and transcatheter mitral valve repair.

## Data Availability

The datasets generated and analysed during the current study are not publicly available due to the institution policy but are available from the corresponding author on reasonable request.

## Supplementary Material

Supplementary material associated with this article can be found, in the online version, at https://doi.org/10.31083/j.rcm2507241.
