# Supplementary material for: Association of Mitral Valve Geometry at CT with Secondary Mitral Regurgitation after Transcatheter Aortic Valve Replacement in Patients with Aortic Regurgitation
Source: Rev Cardiovasc Med. 2024 Jul 2;25(7):241. doi: 10.31083/j.rcm2507241 (PMC11317326; doi:10.31083/j.rcm2507241)
Supplement: Supplementary file 1 [file 2153-8174-25-7-241-s1.docx]

Supplementary Table 1. Reproducibility analysis

| Intraclass correlation coefficients test | Intraobserver variation | Interobserver variation |
| --- | --- | --- |
| Trigone-to-trigone distance | 0.95(0.88-0.98) | 0.89(0.75-0.96) |
| Anteroposterior distance | 0.99(0.96-0.99) | 0.98(0.95-0.99) |
| Intercommisural distance | 0.97(0.92-0.99) | 0.93(0.83-0.97) |
| Annulus area | 0.99(0.96-0.99) | 0.95(0.89-0.98) |
| Annulus perimeter | 0.99(0.97-0.99) | 0.95(0.88-0.98) |
| Mitral valve tenting height | 0.91(0.80-0.97) | 0.86(0.69-0.94) |
| Mitral valve tenting area | 0.90(0.76-0.96) | 0.88(0.72-0.95) |
| ALPM-MA distance | 0.96(0.90-0.98) | 0.93(0.84-0.97) |
| PMPM-MA distance | 0.93(0.84-0.97) | 0.89(0.73-0.95) |
| PM-PM distance | 0.95(0.89-0.98) | 0.91(0.78-0.96) |

ALPM-MA: anterolateral papillary muscle to mitral annulus; PMPM-MA: posteromedial papillary muscle to mitral annulus; PM-PM: papillary muscle head to papillary muscle head.

Supplementary Table 2. Univariable logistic regression to predict persistent mitral regurgitation after transcatheter aortic valve replacement

| Variables | Odds ratio (95% confidence interval) | *p* valve |
| --- | --- | --- |
| Age (years) | 0.99 (0.92, 1.06) | 0.76 |
| Female | 1.20 (0.44, 3.30) | 0.72 |
| Hypertension | 0.45 (0.14, 1.40) | 0.17 |
| COPD | 3.07 (0.79, 11.87) | 0.11 |
| Coronary artery disease | 1.22 (0.36, 4.20) | 0.75 |
| Atrial fibrillation | 3.18 (1.02, 9.90) | 0.046 |
| QRS duration>120ms | 3.20 (1.07, 9.57) | 0.04 |
| LA (mm) | 1.08 (1.00, 1.16) | 0.06 |
| LVESD (mm) | 1.05 (1.00, 1.11) | 0.054 |
| LVEF (%) | 0.97 (0.93, 1.01) | 0.09 |
| Anteroposterior distance(mm) | 1.25 (1.07, 1.46) | 0.005 |
| Intercommisural distance(mm) | 1.16 (1.03, 1.32) | 0.02 |
| Annulus area(cm^2^) | 1.47 (1.13, 1.92) | 0.004 |
| Annulus perimeter(mm) | 1.07 (1.02, 1.12) | 0.005 |
| Mitral valve tenting height (mm) | 1.66 (1.23, 2.24) | 0.001 |
| Mitral valve tenting area (cm^2^) | 8.71 (2.25, 33.79) | 0.002 |
| PM-PM distance (mm) | 1.10 (1.00, 1.21) | 0.06 |

Values are odds ratio (95% confidence interval). COPD: chronic obstructive pulmonary disease; LA: left atrial dimension; LVESD: left ventricular end-systolic dimension; LVEF: left ventricular ejection fraction; PM-PM: papillary muscle head to papillary muscle head.
